# The rural bite in population pyramids: what are the implications for responsiveness of health systems in middle income countries?

**DOI:** 10.1186/1471-2458-14-S2-S8

**Published:** 2014-06-20

**Authors:** Nowrozy Kamar Jahan, Pascale Allotey, Dharma Arunachalam, Shajahan Yasin, Ireneous N Soyiri, Tamzyn M Davey, Daniel D Reidpath

**Affiliations:** 1 South East Asia Community Observatory (SEACO), Monash University, Segamat Johor, Malaysia; 2 Global Public Health, JC School of Medicine and Health Sciences, Monash University, Malaysia; 3 Political and Social Inquiry, Faculty of Arts, Monash University, Australia

## Abstract

**Background:**

Health services can only be responsive if they are designed to service the needs of the population at hand. In many low and middle income countries, the rate of urbanisation can leave the profile of the rural population quite different from the urban population. As a consequence, the kinds of services required for an urban population may be quite different from that required for a rural population. This is examined using data from the South East Asia Community Observatory in rural Malaysia and contrasting it with the national Malaysia population profile.

**Methods:**

Census data were collected from 10,373 household and the sex and age of household members was recorded. Approximate Malaysian national age and sex profiles were downloaded from the US Census Bureau. The population pyramids, and the dependency and support ratios for the whole population and the SEACO sub-district population are compared.

**Results:**

Based on the population profiles and the dependency ratios, the rural sub-district shows need for health services in the under 14 age group similar to that required nationally. In the older age group, however, the rural sub-district shows twice the need for services as the national data indicate.

**Conclusion:**

The health services needs of an older population will tend towards chronic conditions, rather than the typically acute conditions of childhood. The relatively greater number of older people in the rural population suggest a very different health services mix need. Community based population monitoring provides critical information to inform health systems.

## Background

Demographic and epidemiological transitions are necessarily correlated. The demographic profile of a country indicates the risk groups across age, sex or ethnicity related risk factors. The demographic profile therefore is an important indicator to inform disease priorities. Most low and middle income countries for instance, have a classic expansive population pyramid which depicts a 'youth bulge' or a greater proportion of younger people. This usually indicates a high fertility rate and therefore a greater need for reproductive health programs and infant and child health services [1]. Higher income countries have stationary or more commonly, constrictive population pyramids which indicate similar proportions across ages, or negative growth and aging populations respectively [2]. Countries with a higher proportion of older people will require a proportionately greater investment in health services for the aged, catering particularly for the chronic non-communicable diseases (NCDs) prevalent in later life. Demographic information is therefore critical to priority setting for preventive, promotive, curative and rehabilitative health care services and the allocation of resources to meet the needs of the various sectors of the population.

Demographic transitions monitor the changes in the population profiles over time, providing an indicator of economic and industrial development. Also important is the monitoring of the distribution of populations across urban and rural areas as an indication of urbanisation and internal migration patterns. As a strategy for implementing development agendas, urbanisation has been a consistent trend in low and middle income countries. A World Bank publication highlights that “no country has ever reached middle-income status without a significant population shift into cities” [3]. For individuals the move to urban centers is often driven by family interests or a personal desire to seek one's fortune where there is a greater diversity of opportunities and greater earning potential [4-6].

There has been significant research undertaken on the effects of urban growth and the implications for health systems and health care [7-9]. However, relatively little is known about how well health systems adjust to urbanisation with respect to the provision of services to rural communities, particularly in the context of low and middle income countries. If for instance, the migration of people away from rural areas were represented proportionately across the age groups then the demographic profile of the rural community would remain exactly the same before and after migration. The population would have shrunk, but the demographic profile of the population would remain constant. The required health service mix would remain the same, with an expectation of a diminished demand given the reduced population. The actual pattern of rural to urban migration, however, varies across different countries reflecting the drivers of rural urban migration. In some countries in sub-Saharan Africa, the highly gendered nature of urbanisation means that men tend to move for work but continue to visit their families in the rural communities. Fertility rates therefore do not decline significantly [10] and reproductive health services continue to be critical. At the same time reviews on population trends indicate a growing elderly population in rural communities in developing countries [11] highlighting a need for elderly care. Data from the US suggests that the rural-flight is predominantly by both male and female young adults leaving the family home [12].

As an aspiring middle income country in Southeast Asia, Malaysia has an explicit policy to promote rural-to-urban migration as part an industrialisation/development agenda [13]. Like most middle income countries, it is undergoing an epidemiological transition and records one of the highest rates of obesity and diabetes in the region [13]. In addition, it has also maintained one of the higher fertility rates for an upper middle income country – above the 75^th^ percentile. This mix creates additional health systems challenges with respect to the appropriate mix of service provision across a country that is highly urbanised but nonetheless retains a significant rural population. Using census data from the South East Asia Community Observatory (SEACO), a rural health and demographic research surveillance system in Segamat, Johor, Malaysia, this paper compares the rural and national population profiles and discusses the implications for health systems and health care.

## Methodology

A comparative analysis is made between the estimated Malaysian national mid-year population profile for 2012, and the equivalent population profile for SEACO. SEACO is a health and demographic surveillance site located in the district of Segamat, in the state of Johor on the southern tip of the Malay Peninsula [14]. The district of Segamat comprises urban, rural and plantation areas with an ethnic mix (Malays, Chinese and Indian) close to national proportions. SEACO operates within five mukim or sub-districts: Bekok, Chaah, Gemereh, Jabi, and Sungai Segamat.

Between March 2012 and February 2013 census collectors visited 14,353 houses. Approximately 11 percent of houses were vacant. Of the occupied houses, basic demographic information was collected from 10,373 household (81%). Basic demographic data was collected on 38,228 people (50.3% male and 49.6% females).

### Data sources

The estimated national mid-year population for Malaysia, broken down by five year age groups and sex was obtained from the US Census Bureau's International Database for 2012 [15]. The total population was an estimated 29.1 Million people (50.8% male and 49.2% female).

The sub-district population data were derived from the census round of SEACO, a newly established health and demographic surveillance system (HDSS) [16]. The data were broken down by five year age group, sex, and citizenship.

The child, aged and total dependency ratios (and their inverses, support ratios) are used to contrast the national and sub-district population profiles.

The total dependency ratio is the sum of the aged and the child population in the numerator. Population pyramids are used as points of visual contrast.

### Ethics

The SEACO health and demographic surveillance system was launched by the Chief Minister for Johor state in 2011 and established with the approval of the Monash University Human Research Ethics Committee (MUHREC CF11/3663 - 2011001930).

## Results

**Figure 1:**
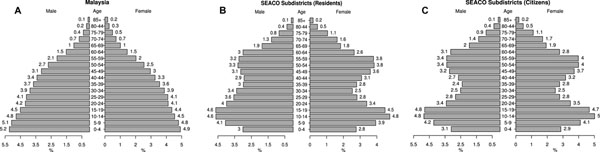
Population pyramids for (A) Malaysia, (B) the SEACO sub-district, (C) Malaysian citizens in the SEACO sub-district

Nationally the population pyramid has the classic expansive population pyramid shape of a country with a relatively high fertility rate (Figure 1A). It does not, however, display the rapid tailing-off of the population as it ages – typical of developing countries. Furthermore the profile shows the development of an aging population. The SEACO sub-district population pyramids are best considered together (Figure 1B and 1C). The pyramids show a larger teenage than under 10 population, suggesting a declining demand for maternal and child health (MCH) services. From 20 years of age there is a sharp decline in the population, gradually increasing again from 40 years of age and peaking in the 55-59 year age group. The decline in the young adult population is a function of “rural-flight”. On completion of schooling, people 20 years and over leave the district for employment, education and marriage. The effect seems to be stronger for females than males in the population pyramid of residents (Figure 1B), but similar in the population pyramid of citizens (Figure 1C). The arrival of Indonesian plantation workers, shown in Figure 1B, reduces to some extent the loss of males in the 20 to 40 age group, but has little or no impact on the female population.

Figure 1 shows three different population pyramids. The first is the Malaysia national population pyramid based on the US Census Bureau estimates (A), the second is the SEACO sub-district population pyramid counting all residents (B), and the third is the SEACO sub-district population pyramid counting only the Malaysian nationals (citizens) (C). The sub-district population pyramids are shown separately for all residents and citizens because Segamat has a significant, and almost exclusively male, agricultural sector employing largely Indonesian workers. The effect of the Indonesian workers on the health systems needs to be considered separately.

The proportion of the sub-district population in the 60-64, 65-69 and 70-74 year age groups is twice that of the national data.

The aged, child and total dependency ratios show the size of the dependent population relative to the working age population. Table 1 shows the dependency ratios for Malaysia and for the population of the SEACO sub-district.

**Table 1:** Aged and child dependency ratios for Malaysia and the SEACO sub-district

	National Data		Sub-district Data	
	Dependency Ratio	Support Ratio	Dependency Ratio	Support Ratio
Aged Dependency	0.078	12.81	0.147	6.83
Child Dependency	0.448	2.23	0.37	2.7
Total Dependency	0.526	1.90	0.517	1.93

The aged dependency ratio for the SEACO sub-district is almost twice the national ratio. There are 6.8 productive residents in the sub-district for every person aged 65 and over; and there are 12.8 productive residents nationally for every person aged 65 and over. The child dependency ratios at the national and sub-district level are much closer in value, with the SEACO sub-district having a slightly lower ratio; i.e., fewer child dependents per productive resident.

The difference in where the dependency lies can also be examined by considering the ratio of child dependency to aged dependency. Nationally, the ratio is 5.74. That is, there are 5.74 children aged 0-14 for every adult aged 65 and over. In the SEACO sub-district, there are less than half as many children aged 0-14 as there are adults aged 65 and over (2.53).

## Discussion

The population profile from the rural, sub-district census is radically different from the national profile. With very similar child dependency ratios, and much larger aged dependency ratios to the national average, one could anticipate a similar or reduced need for MCH services, but twice the need for aged services. The indication of a reducing need for MCH services is further reinforced by the declining proportion of young children in the population compared with the national average. In the SEACO sub-district there were twice as many aged dependents to child dependents as there were nationally.

Assuming that the proportion of 0-4 year olds continues to decline in the SEACO sub-district, and continued rural-flight for adults in the 20-25 year age group, over the next 15 years the aging of the sub-district will become even more stark, with the health service needs of the district being almost entirely related to the management of chronic NCDs and aged care.

The health services requirement for the 0-14 year age group are concentrated in the first two years of life – and the antenatal period for pregnancy care. Health services are therefore intensive for maternal health and the under 5 year population. Demand then declines rapidly, to a low level requirement to maintain immunisation protocols and occasional acute care for injuries and infectious diseases. The pattern of health service utilisation with aging is one of a rapidly increasing need for chronic and acute care. A 2011 national health survey showed that 30% of people aged over 65 had diabetes compared with 11% and 18% in the 35-39 and 40-44 year age groups respectively [17]. For hypertension it was 70% compared with 27% and 36%. For hypercholesterolaemia it was 55% compared with 35% and 41%. Not only are the risk factors for heart disease, stroke, kidney disease, and blindness increasing rapidly with age, it is the chronic exposure to the risk factors that result in the need for acute care and management. Aging populations are populations that can anticipate a heavy demand for health services.

Furthermore studies in migration more broadly highlight the healthy migrant effect [20,21,23]. This describes the pattern of migration where the healthiest young adults leave the rural areas. The implication is that there is an increasingly unhealthy rural population [12,22]. What is unclear are the health needs of those who remain and the implications of the health profile of residents for the health systems. This is an important research gap.

The obvious limitation of this study is its reliance on census data from one rural area and this raises questions about whether another rural area would show similar patterns. It seems unlikely that the SEACO sub-district would be radically different from other rural areas in peninsular Malaysia, particularly given the explicit government policy to encourage working aged populations to move into urban centres [23]. Even if it were different, however, the difference merely highlight the need for health systems to be flexible enough to cater for the needs of the population and anticipate the future needs. Health services delivered through a tiered system of tertiary, secondary and primary services, where secondary services are district based and primary services are sub-district based, cannot rely on National profiles and need to respond to population specific demands.

## Conclusion

Low and middle income countries have traditionally focused on maternal and child health as their core deliverable reflecting an expansive population pyramid. Within the Southeast Asia region, countries like Malaysia increasingly recognise the growing requirement to support NCDs management [25]. However these services are not yet targeted to take account of urbanisation and internal migration. Significant rural to urban migration can complicate the service provision issues. For Ministries of Health to be appropriately responsive to health systems, they need to monitor and take into account current data on the demographic profiles of populations.

## Competing interests

The authors have no competing interests.

## Authors' contributions

Reidpath and Jahan discussed and produced the initial draft of the paper. Further revisions were undertaken by Allotey, Arunachalam, Yasin, Soyiri and Davey towards the final version of the manuscript.

## Declarations

Funding for SEACO is provided by the Research Offices of Monash University campuses in Australia and Malaysia; the Faculty of Medicine, Nursing and Health Sciences; the Jeffrey Cheah School of Medicine and Health Sciences; and the Faculty of Arts.

This article has been published as part of *BMC Public Health* Volume 14 Supplement 2, 2014: Responsive health systems: working with the community on control of non-communicable diseases (NCDs). The full contents of the supplement are available online at http://www.biomedcentral.com/bmcpublichealth/supplements/14/S2.
